# The taccalonolides and paclitaxel cause distinct effects on microtubule dynamics and aster formation

**DOI:** 10.1186/1476-4598-13-41

**Published:** 2014-02-28

**Authors:** April L Risinger, Stephen M Riffle, Manu Lopus, Mary A Jordan, Leslie Wilson, Susan L Mooberry

**Affiliations:** 1 Department of Pharmacology, University of Texas Health Science Center at San Antonio, San Antonio, TX 78229, USA; 2 Cancer Therapy & Research Center, University of Texas Health Science Center at San Antonio, San Antonio, TX 78229, USA; 3 Department of Molecular, Cellular and Developmental Biology, University of California, Santa Barbara, CA 93106, USA; 4 UM-DAE Centre for Excellence in Basic Sciences, University of Mumbai Campus, Kalina, Santacruz East, Mumbai 400098, India

**Keywords:** Taccalonolide, Microtubule, Paclitaxel, Microtubule stabilizer, Tubulin

## Abstract

**Background:**

Microtubule stabilizers suppress microtubule dynamics and, at the lowest antiproliferative concentrations, disrupt the function of mitotic spindles, leading to mitotic arrest and apoptosis. At slightly higher concentrations, these agents cause the formation of multiple mitotic asters with distinct morphologies elicited by different microtubule stabilizers.

**Results:**

We tested the hypothesis that two classes of microtubule stabilizing drugs, the taxanes and the taccalonolides, cause the formation of distinct aster structures due, in part, to differential effects on microtubule dynamics. Paclitaxel and the taccalonolides suppressed the dynamics of microtubules formed from purified tubulin as well as in live cells. Both agents suppressed microtubule dynamic instability, with the taccalonolides having a more pronounced inhibition of microtubule catastrophe, suggesting that they stabilize the plus ends of microtubules more effectively than paclitaxel. Live cell microscopy was also used to evaluate the formation and resolution of asters after drug treatment. While each drug had similar effects on initial formation, substantial differences were observed in aster resolution. Paclitaxel-induced asters often coalesced over time resulting in fewer, larger asters whereas numerous compact asters persisted once they were formed in the presence of the taccalonolides.

**Conclusions:**

We conclude that the increased resistance of microtubule plus ends to catastrophe may play a role in the observed inability of taccalonolide-induced asters to coalesce during mitosis, giving rise to the distinct morphologies observed after exposure to these agents.

## Background

While microtubule targeting agents are among the most effective therapies used in cancer treatment, little is known about their mechanisms of action downstream of inhibiting microtubule dynamics. One confounding issue is that microtubule stabilizers, including paclitaxel, have diverse cellular effects depending on the concentrations used. In cell culture, low concentrations of paclitaxel (5–10 nM) are sufficient to slow microtubule dynamics, resulting in an extended mitotic delay that cells can eventually exit, albeit often with misaligned chromosomes [1]. These chromosomal defects can lead to cell death following mitotic slippage or aberrant division. Higher concentrations of paclitaxel (10 to 500 nM) cause a concentration-dependent increase in the duration of mitotic arrest [2] and the formation of abnormal mitotic spindles or asters, some of which are not nucleated by a centrosome. These multiple asters prevent normal mitosis and lead to apoptosis during mitosis or shortly after mitotic exit [3]. Interestingly, substantially higher, micromolar concentrations of paclitaxel are required to cause bundling of interphase microtubules, which is the most recognizable phenotype associated with this class of drugs [4,5].

Recent studies have shown that paclitaxel exhibits these same concentration dependent effects *in vivo* when visualized by intravital microscopy [6]. A sub-therapeutic dose of 1.2 mg/kg extended the duration of mitosis in dividing tumor cells. These cells formed bipolar spindles, often with chromosomal alignment defects, and they eventually completed mitosis. However, slightly higher, antitumor concentrations of paclitaxel caused dividing tumor cells to arrest in mitosis, often with multiple asters [6]. Although recent studies clearly indicated that the antitumor actions of microtubule targeted agents also involved their effects on interphase cells [7,8], the finding that the formation of multiple microtubule asters is closely correlated with effective antitumor doses of paclitaxel suggested that a more detailed analysis of this process may inform the antitumor mechanisms of microtubule stabilizers, including paclitaxel.

Taccalonolides A and E, the most prevalent taccalonolides isolated from plants of the genus *Tacca,* cause microtubule bundling with short thick tufts of microtubules that appear quite different from paclitaxel-induced interphase microtubule bundles [4,9]. These taccalonolides are also distinct from paclitaxel because they circumvent multiple mechanisms of drug resistance, including P-glycoprotein mediated resistance both *in vitro* and *in vivo*[9,10] as well as mutations in the taxane binding site [9] and in cells expressing βIII tubulin [10]. At the cellular level, taccalonolide A is highly persistent following short periods of exposure to the drug, an effect that is different from most microtubule stabilizers [4]. While taccalonolides A and E cause dramatic microtubule stabilization in cells and have antitumor effects *in vivo*, no direct interaction with microtubules was observed, possibly because these agents are significantly less potent than other microtubule stabilizers [11]. Recently, potent taccalonolides have been isolated and generated by semi-synthesis [12]. The taccalonolides AF and AJ differ from taccalonolide A and B by the incorporation of a C22,C23 epoxide moiety. They have potencies in the range of other microtubule stabilizers, including paclitaxel, and demonstrate for the first time the ability of a taccalonolide to interact directly with tubulin/microtubules through a covalent interaction [13]. Cellular data suggest that these potent taccalonolides have the same cellular properties as taccalonolide A, including strong persistence, the propensity to cause microtubule bundling at low antiproliferative concentrations and their effects on cellular signaling and centrosomal separation, which differ from either paclitaxel or laulimalide [4,14]. Therefore, the epoxidation of C22,23 appears to dramatically increase the potency of the taccalonolides in biochemical and cellular assays while retaining the mechanisms of action of less potent taccalonolides.

Microtubule stabilizers, including each of the taccalonolides, cause the formation of abnormal mitotic cells with multiple microtubule asters and cause the mitotic arrest and death of cancer cells [9]. However, the structures of the asters formed by the taccalonolides are markedly different from those induced by members of the two major classes of microtubule stabilizers, paclitaxel or laulimalide [14]. At concentrations that cause mitotic arrest, paclitaxel often results in the formation of one diffuse aster with two smaller punctate asters. In contrast, treatment with taccalonolides causes the appearance of much more numerous, compact asters [9,14].

The goal of this study was to compare the effects of paclitaxel and taccalonolide AJ on parameters of dynamic instability, both in biochemical preparations and in live cells. We hypothesized that the different effects of microtubule stabilizers on microtubule dynamics might play a role in the formation of distinct microtubule asters. Our results show that the distinct ability of the taccalonolides to inhibit microtubule shortening and catastrophe might partially explain the inability of multiple microtubule asters to coalesce, giving rise to the distinct mitotic microtubule morphology that has been observed for this class of drugs.

## Results

### Effects of taccalonolide AJ and paclitaxel on the dynamic instability of purified tubulin

The effects of the taccalonolides on multiple parameters of dynamic instability were measured at the plus ends of steady-state microtubules made from phosphocellulose-purified tubulin seeded from axonemes and compared to the effects caused by paclitaxel. For these studies the semi-synthetic taccalonolide AJ was used because it has been shown to stimulate microtubule polymerization in biochemical assays [12].

Similar to the effects of other microtubule stabilizing drugs, taccalonolide AJ suppressed microtubule dynamics at concentrations sub-stoichiometric to tubulin (Table 1). Taccalonolide AJ had no significant effect on the plus-end growth rate; however, it suppressed the shortening rate in a concentration-dependent manner, with inhibition of 38% and 66% at 1 and 3 μM, respectively. Additionally, taccalonolide AJ inhibited both the catastrophe frequency and the rescue frequency in a concentration-dependent manner. Specifically, at 1 μM taccalonolide AJ, a 25% reduction in the catastrophe frequency and a 29% reduction in the rescue frequency were measured. At 3 μM, more extensive inhibition of both the catastrophe and rescue frequencies of 54% and 44%, respectively occurred (Table 1). Taccalonolide AJ-induced suppression of shortening rate, catastrophe and rescue frequencies resulted in concentration-dependent decreases in growing and shortening time with a concomitant increase in the time microtubules were in an attenuated state. Taccalonolide AJ at 1 μM caused a 104% increase in the attenuated state and a 36% decrease in overall dynamicity of the microtubules. At 3 μM, a 148% increase in attenuation was measured and the overall dynamicity of microtubules was suppressed by 65%. These results show that taccalonolide AJ, similar to other microtubule stabilizers, directly suppresses the dynamic instability of microtubules in a concentration-dependent manner.

**Table 1:** Phosphocellulose purified bovine brain microtubules were assembled to steady state in the presence of taccalonolide AJ, paclitaxel or vehicle and the dynamic instability parameters were determined

	**Vehicle**	**1 μM AJ**		**3 μM AJ**		**250 nM PTX**	
	**Value ± SEM**	**Value ± SEM**	**% Change**	**Value ± SEM**	**% Change**	**Value ± SEM**	**% Change**
Growth rate (μm/min)	2.7 ± 0.2	3.1 ± 0.2	+15	3 ± 0.3	+11	2.8 ± 0.2	+4
Shortening rate (μm/min)	11.9 ± 1	7.4 ± 0.6^**^	−38	4 ± 0.5^***^	−66	5.8 ± 0.4^***^	−51
Percent time growing	56	34	−39	26	−54	26	−54
Percent time shortening	19	15	−21	12	−37	22	+16
Percent time attenuated	25	51	+104	62	+148	52	+108
Catastrophe frequency (per min)	0.28 ± 0.05	0.21 ± 0.04^*^	−25	0.13 ± 0.03^***^	−54	0.26 ± 0.04	−7
Rescue frequency (per min)	0.84 ± 0.17	0.60 ± 0.15	−29	0.47 ± 0.15^*^	−44	0.30 ± 0.09^**^	−64
Dynamicity (μm/min)	2.73	1.74	−36	0.95	−65	1.63	−40

Approximately 25 microtubules were measured for each condition.

^*^P<0.05; ^**^P < 0.01; ^***^P < 0.001, as determined with a Student’s *t*-test.

The effects of taccalonolide AJ on suppression of most parameters of microtubule dynamic instability occurred in a manner similar to paclitaxel, albeit less potently. The same degree of dynamicity inhibition (−36% vs. -40%) was induced by 250 nM paclitaxel and 1 μM taccalonolide AJ, respectively. Paclitaxel and taccalonolide AJ both suppressed the microtubule plus-end shortening rate with 51% inhibition by 250 nM paclitaxel and only 38% inhibition by 1 μM taccalonolide AJ (Table 1). The percent growing time was inhibited by both compounds, but taccalonolide AJ also inhibited the percent shortening time which was slightly increased by paclitaxel. Both drugs increased the fraction of time that the microtubules remained in the attenuated state, although paclitaxel was again more potent because 250 nM paclitaxel increased the time attenuated by 108% with 1 μM taccalonolide AJ causing a 104% increase.

Although taccalonolide AJ and paclitaxel exhibited many of the same effects on microtubule dynamics, there were major differences observed in the frequency of catastrophe and rescue. Whereas 250 nM paclitaxel did not significantly alter the catastrophe frequency, 1 μM taccalonolide AJ suppressed catastrophe frequency by 25% (Table 1). In contrast, 250 nM paclitaxel caused a large, 64%, decrease in rescue frequency, while no significant effect on this parameter was caused by 1 μM taccalonolide AJ. At these same concentrations, the two drugs caused equivalent effects on overall microtubule dynamicity, preferentially suppressing either the catastrophe or rescue frequencies, respectively. The greater suppression of the catastrophe frequency caused by taccalonolide AJ indicated that the microtubules have a lower propensity to revert to a shrinking state compared to paclitaxel. In contrast, paclitaxel has a larger effect on the suppression of rescue frequency and thus a higher propensity than taccalonolide AJ to inhibit the switch to microtubule growth.

### Effects of Taccalonolide AJ and paclitaxel on microtubule dynamics in live cells

The effects of paclitaxel and taccalonolide AJ on microtubule dynamics in MCF7-EGFP-α-tubulin cells were also evaluated (Table 2). Taccalonolide AJ and paclitaxel were found to have nearly identical antiproliferative potencies in this cell line with IC_50_ values of 37 ± 4.6 nM and 36 ± 3.8 nM, respectively (data not shown), allowing for a direct comparison of equal drug concentrations on microtubule dynamics.

**Table 2:** Suppression of microtubule dynamic instability in MCF7-EGFP-α-tubulin cells incubated with vehicle, taccalonolide AJ or Paclitaxel

	**Vehicle**	**25nM AJ**		**50nM AJ**		**100nM AJ**		**50nM Paclitaxel**		**150nM Paclitaxel**	
	**Value ± SEM**	**Value ± SEM**	**% Change**	**Value ± SEM**	**% Change**	**Value ± SEM**	**% Change**	**Value ± SEM**	**% Change**	**Value ± SEM**	**% Change**
Growth rate (μm/min)	7.5±0.25	7.2±0.3	−5.1	6.9±0.3	−8.50	6.5±0.3	−13.3^**^	7.2±0.3	−3.9	6.5± 0.3	−13.3^**^
Shortening rate (μm/min)	13.9±0.9	12.0±0.8	−14.0	8.6±0.4	−38.5^***^	9.1±0.9	−34.9^***^	12.0±0.7	−13.9	8.0±0.5	−42.5^***^
Percent Time Growing	35.8	29.2	−18.6	14.9	−58.3	14.9	−58.4	26.0	−27.5	18.2	−49.3
Percent Time Shortening	16.6	13.7	−17.3	9.7	−41.6	9.7	−41.7	11.5	−31.1	7.8	−53.4
Percent Time Attenuated	47.6	57.1	20.1	75.4	59.0	75.4	58.6	62.6	31.6	74.1	55.8
Catastrophe Frequency (per min)	1.8±0.1	1.6±0.2	−8.9	1.2±0.1	−32.8^***^	1.2±0.1	−36.1^***^	1.4±0.1	−21.1^*^	1.3±0.2	−30.0^*^
Rescue Frequency (per min)	10.4±0.5	10.5±0.6	0.6	11.6±0.7	11.4	11.0±0.7	5.9	11.2±0.6	7.5	12.8±0.6	22.8^**^
Dynamicity	5.1	3.8	−25.3	1.9	−62.3	1.9	−62.1	3.3	−34.1	1.9	−62.33
MTs/Cells Counted	40/18	41/13		41/13		31/12		47/14		30/13	
% Dynamic MTs	65	51		41		32		54		44	

^*^P < 0.05; ^**^P < 0.01; ^***^P < 0.001 as determined with a Student’s *t*-test.

Similar to its effects on purified tubulin, taccalonolide AJ caused dose-dependent suppression of microtubule dynamics over a range of 25 – 100 nM (Table 2). The most dramatic effects of taccalonolide AJ were a marked suppression of both the shortening rate and the catastrophe frequency, which were suppressed at 50 nM by 39% and 33%, respectively, and were not suppressed further at higher concentrations. These changes led to decreases in overall dynamicity of 25% and 62% at 25 and 50 nM, respectively (Table 2). The almost identical effects observed at 50 and 100 nM taccalonolide AJ for all parameters suggested that drug binding or uptake may be saturated at 50 nM over the 4-h time course.

Paclitaxel caused a similar dose-dependent suppression of shortening rate and catastrophe frequency; however, higher concentrations of paclitaxel than of taccalonolide AJ were required to observe comparable effects. For instance, overall dynamicity was decreased 34% by 50 nM paclitaxel while the same concentration of taccalonolide AJ caused a 62% decrease (Table 2). An almost identical decrease in overall dynamicity was observed for 150 nM paclitaxel versus 50 nM taccalonolide AJ, demonstrating that taccalonolide AJ is a more potent inhibitor of microtubule dynamics than paclitaxel in live cells, although the reverse was true with purified tubulin (Tables 1 and 2).

At concentrations of 50 nM taccalonolide AJ and 150 nM paclitaxel, which caused a similar suppression of overall dynamicity, many of the individual parameters of dynamic instability were also identical, including growth and shortening rates and catastrophe frequency (Table 2). However, one distinct finding was that 150 nM paclitaxel significantly increased the rescue frequency by 23%, which was not affected at any concentration of taccalonolide AJ. Together, these data demonstrate that taccalonolide AJ decreases microtubule dynamicity similarly to paclitaxel, but with notable distinctions in the frequency of rescue and catastrophe that might impart differences in their cellular effects.

### Differential mitotic microtubule structures formed by the taccalonolides and paclitaxel

Microtubule targeting agents arrest cancer cells in mitosis with abnormal mitotic microtubule asters. Distinct microtubule morphologies are induced by different microtubule stabilizers, particularly paclitaxel and the taccalonolides [9,14]. We hypothesized that their subtle differences on the inhibition of microtubule dynamics contribute to the formation of morphologically distinct mitotic asters. Since these drugs range in potency, we compared the effects of the taccalonolides and paclitaxel on spindle formation at the minimum concentration that caused maximal mitotic arrest.

**Figure 1:**
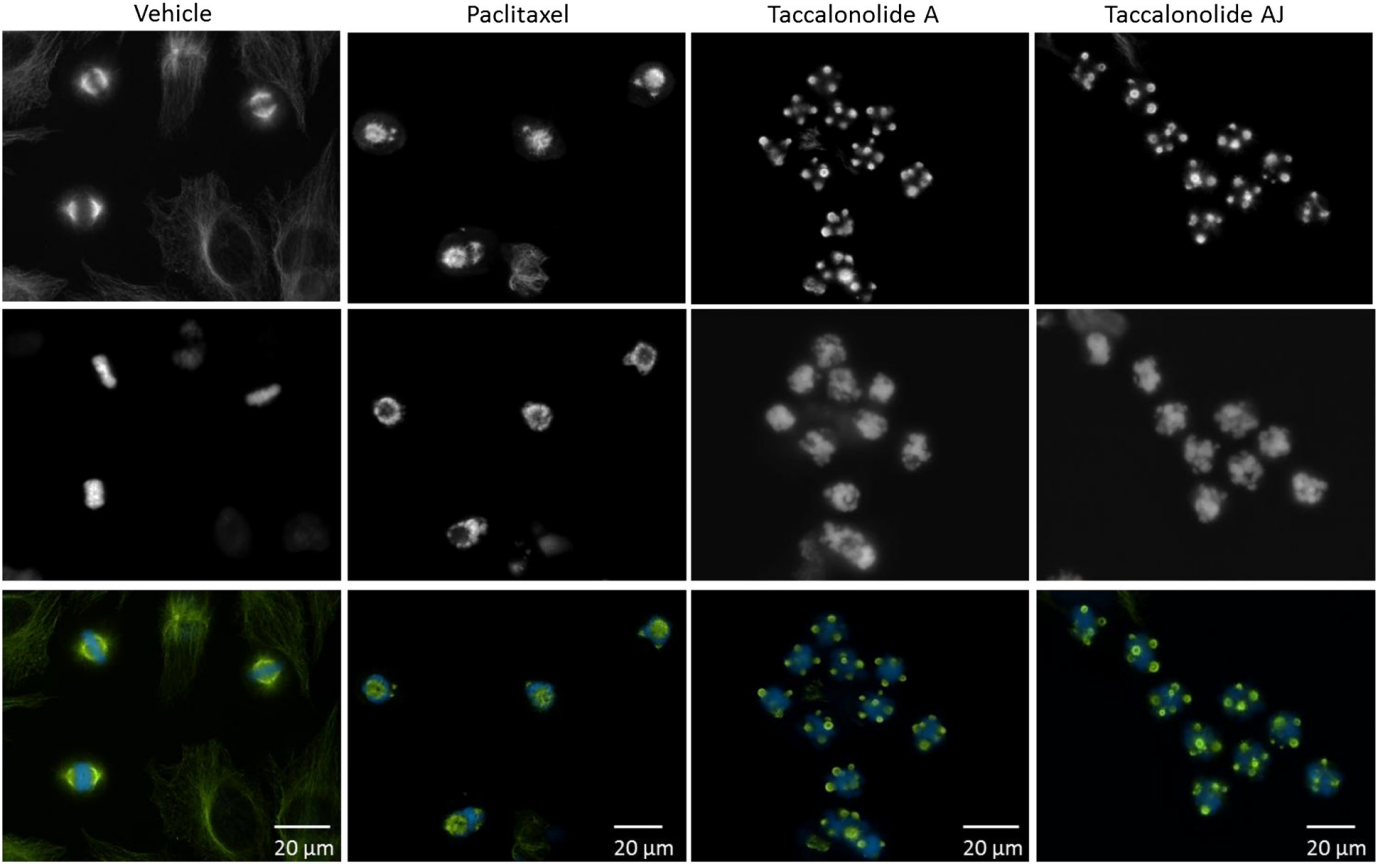
**Mitotic asters induced by paclitaxel or the taccalonolides.** The mitotic asters in HeLa cells treated with vehicle, 12 nM paclitaxel, 5 μM taccalonolide A or 20 nM taccalonolide AJ, the minimum concentrations that caused maximum mitotic accumulation, were visualized by indirect immunofluorescence for β-tubulin (top). DNA was visualized by DAPI staining (middle) and merged images are shown with microtubules in green and DNA in blue (bottom).

The lowest concentration that caused maximal G_2_/M arrest of HeLa cells was determined for each drug using flow cytometry and was found to be 12 nM for paclitaxel, 20 nM for taccalonolide AJ and 5 μM for taccalonolide A. Consistent with prior results, the phenotypes of the mitotic asters formed by these drugs were quite different (Figure 1). In comparison to vehicle-treated cells that contained a normal bipolar spindle (Figure 1), the majority of paclitaxel-treated cells contained a single large, diffuse aster that was often accompanied by two smaller, more punctate asters (Figure 1). In contrast, cells treated with either taccalonolide A or AJ contained 5–7 asters that were small, compact and consistent in morphology (Figure 1).

### Mechanisms of aster formation identified by live cell microscopy

**Figure 2:**
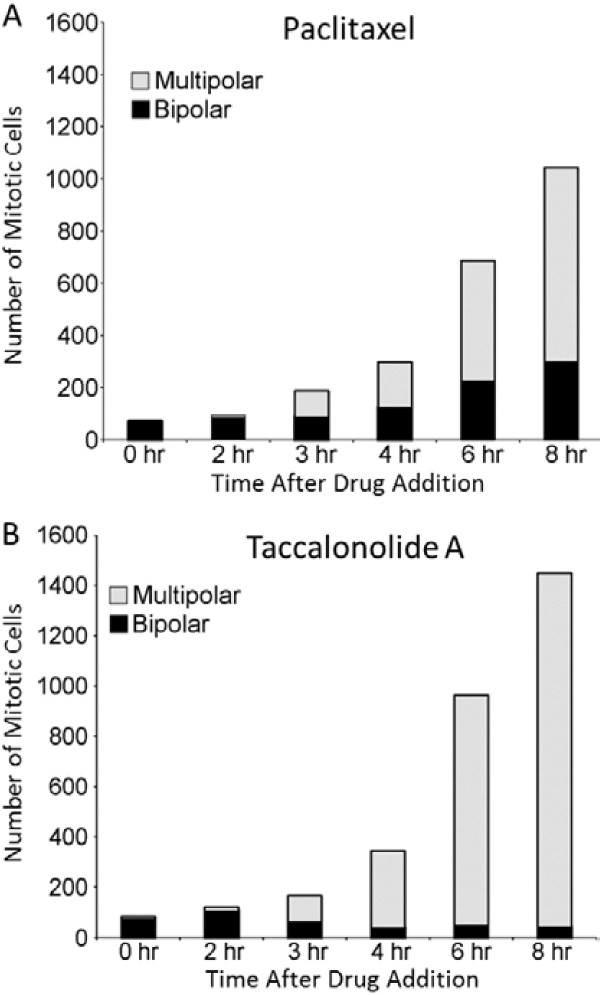
**Effects of paclitaxel or taccalonolide A on mitotic accumulation and aster number.** The number of mitotic cells present at each time after the addition of **(A)** 12 nM paclitaxel or **(B)** 5 μM taccalonolide A to GFP-β-tubulin expressing HeLa cells was counted. These mitotic cells were classified as containing bipolar spindles (black) or multiple asters (gray). Cells from 60 microscopic fields (20x) from 12 individual wells were analyzed for each condition at each time point.

Studies were initiated using high content imaging with GFP-β-tubulin expressing HeLa cells to identify how these structurally distinct microtubule asters are formed in the presence of paclitaxel or the taccalonolides. As in Figure 1, drugs were added at the minimal concentration that caused maximal G2/M accumulation: 12 nM paclitaxel and 5 μM taccalonolide A. Cells in 60 microscopic fields per treatment condition were followed at 30 min intervals over an 8 h period after drug addition. The number of cells in mitosis and the number of microtubule asters in each cell were counted at each time point. In vehicle-treated cells, the cells continued to cycle normally and did not accumulate in mitosis. At the time of drug addition (0 h), an average of 74 mitotic cells (range 64–82) was observed for each treatment group with the vast majority (89–92%) of these mitotic cells containing normal bipolar spindles (Figure 2). Within 2 h of paclitaxel addition, a slight increase in the number of mitotic cells was observed, but the percentage of those with bipolar spindles remained at 90%. The number of cells in mitosis doubled 3 h after paclitaxel addition and approximately half of these mitotic cells contained more than two asters (Figure 2A). The number of mitotic cells continued to increase from 4–8 h and by 8 h, 14 times as many cells were in mitosis compared to the time of paclitaxel addition. The number and the percentage of cells containing aberrant microtubule asters also increased and by 8 h, 71% of the paclitaxel-treated mitotic cells contained more than 2 microtubule asters (Figure 2A).

A similar pattern of accumulation of cells in mitosis and an increase in the percentage of cells with aberrant microtubule structures was observed with taccalonolide A, with the majority of taccalonolide-treated mitotic cells containing greater than two asters as early as 3 h after drug addition (Figure 2B). By 4 h, 89% of the taccalonolide- treated mitotic cells had more than two asters and the percentage increased to 97% by 8 h. Interestingly, the total number of mitotic cells present after treatment with either agent was similar through the first 3 h, but at 4, 6 and 8 h after treatment more mitotic cells were observed with taccalonolide A treatment than with paclitaxel (Figure 2). While each of the microtubule stabilizers caused accumulation of cells in mitosis with multiple asters, both the total number of cells in mitosis and the percentage of cells containing greater than 2 microtubule asters were substantially higher with taccalonolide treatment, suggesting that the mitotic spindle defects might be more difficult to resolve.

**Figure 3:**
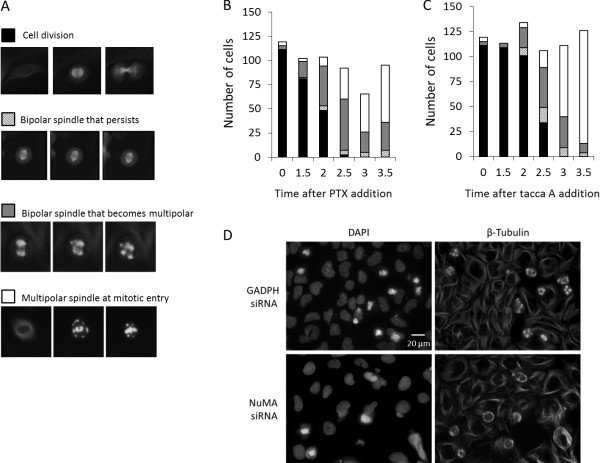
**Aster formation following the addition of paclitaxel or taccalonolide A.** GFP-β-tubulin expressing HeLa cells were treated with 12 nM paclitaxel or 5 μM taccalonolide A. Every cell that entered mitosis within 3.5 h after drug addition was followed until 8 h after drug addition and placed in one of 4 categories. **(A)** Representative images of the 4 categories of cells entering mitosis: bipolar spindle formation followed by cell division, bipolar spindle formation that persisted without completion of mitosis, bipolar spindle that resolved into multiple asters or formation of multiple asters immediately upon mitotic entry. Each image represents approximately 40 μm. The category of cells entering mitosis at the indicated time points following **(B)** paclitaxel or **(C)** taccalonolide A addition are shown. Cells in 60 individual microscopic fields from 12 separate wells were analyzed for each condition at each time point. **(D)** Effects of NuMA depletion on taccalonolide A-induced asters. Microtubules were visualized by indirect immunofluorescence 4 h after the addition of 5 μM taccalonolide A in control (GADPH siRNA) and NuMA-depleted (NuMA siRNA) HeLa cells (right). DNA was visualized by DAPI staining (left).

The same images were analyzed as time courses to observe the formation of mitotic microtubule asters in individual cells following treatment with either paclitaxel or taccalonolide A. The initial microtubule morphology of cells that entered mitosis within 3.5 h after drug addition was noted and followed over the course of 8 h in order to monitor for changes in microtubule structures over time. The fates of cells entering mitosis after drug addition were divided into four categories: 1) cells that entered mitosis with a bipolar spindle followed by cell division, 2) cells that entered mitosis with a bipolar spindle that persisted without any change in spindle or cellular morphology during the observed time course, 3) cells that initially entered mitosis with a bipolar spindle that transitioned into greater than two asters as time progressed and 4) cells that contained 3 or more asters upon entry into mitosis with no indication of passing through a bipolar spindle intermediate. Representative pictures of cells in each of these categories are shown in Figure 3A. The distribution of phenotypes that were observed in cells entering mitosis after paclitaxel or taccalonolide A treatment is presented in Figures 3B and C, respectively. The total number of cells that entered mitosis during each 30 min interval after drug addition was largely unchanged over time, indicating that neither drug had major effects on the rate of mitotic entry.

At the time of drug addition, several cells were in mitosis (time 0). When these cells were followed over the next 8 h, more than 90% completed cell division and no differences were noted between those treated with paclitaxel or taccalonolide (Figure 3B and C, black bars). As early as 1.5 h after paclitaxel addition, the percentage of cells that entered mitosis with bipolar spindles decreased from 93% to 79%. By 2 h after exposure, less than half (47%) of the cells that had entered mitosis were observed to have completed cell division after forming a bipolar spindle (Figure 3B, black bar). After 2.5 h of paclitaxel treatment, only 2% of the cells that entered mitosis were able to form a normal bipolar mitotic spindle and complete cell division. With exposure times of 3 h or longer, no cells of this phenotype were observed.

Cells that entered mitosis with a bipolar spindle that persisted throughout the 8 h total observation period were detected beginning 2 h after paclitaxel treatment (Figure 3B, hatched bar). The number of cells exhibiting this phenotype, 5-8%, remained relatively constant for cells entering mitosis 2–3.5 after paclitaxel addition. Within 2 h of paclitaxel treatment, 40% of the cells entering mitosis contained a bipolar spindle that transitioned into multiple aberrant asters over time (Figure 3B, gray bars). Additionally, 9% of the cells entered mitosis with multiple asters without first forming a bipolar spindle (Figure 3B, white bars). Two to 2.5 h after paclitaxel addition, the number and percentage of cells that entered mitosis with a bipolar spindle that then transitioned into multiple asters increased. At later time points fewer cells exhibited this fate and a concomitant increase in cells that entered mitosis with multiple asters was observed. The percentage of paclitaxel-treated cells exhibiting this phenotype plateaued at 60-62% at later time points (Figure 3B). These results show that paclitaxel caused the formation of multiple types of microtubule structures, including bipolar spindles that were competent for cell division, bipolar spindles that persisted but were dysfunctional, bipolar spindles that transitioned into multiple asters and those that contained more than two asters upon mitotic entry. As time after drug exposure increased, fewer paclitaxel-cells were able to form bipolar spindles upon mitotic entry and those that did form bipolar spindles transitioned to contain multiple asters. At 3.5 h after paclitaxel addition, the majority of the cells entered mitosis with multiple asters that persisted over the remaining period of observation.

Taccalonolide A caused similar effects, with a time-dependent loss of cells that entered mitosis with functional bipolar spindles and a concomitant increase in cells that formed multiple asters after entering mitosis (Figure 3C). However, the kinetics and magnitude of the changes differed between cells treated with paclitaxel or taccalonolide A. The ability of taccalonolide A to induce the formation of dysfunctional spindles was delayed as compared to paclitaxel. At 2 h after taccalonolide A addition, the vast majority of cells, 75%, formed a bipolar spindle and completed cell division. In contrast, less than half of paclitaxel-treated cells treated for 2 h were able to do so. As the time of taccalonolide A exposure increased, fewer cells were able to enter mitosis with a bipolar spindle and 3.5 h after drug addition, no mitotic cells were seen to complete cell division during the course of observation.

Consistent with the effects of paclitaxel, the number and percentage of cells that entered mitosis with bipolar spindles that progressed to multiple asters increased 0–2.5 h after taccalonolide A addition and subsequently decreased at later time points. The number of cells containing multiple asters when they entered mitosis began to increase 2.5 h after drug addition and 90% of cells that entered mitosis 3.5 h after taccalonolide A addition had this phenotype. Interestingly, at the same time point only 63% of paclitaxel-treated cells exhibited multiple asters upon mitotic entry. Later time points, i.e., 4–6 h after drug addition, were evaluated and no further changes in spindle/aster morphologies were detected in either the paclitaxel- or taccalonolide A-treated cells entering mitosis, suggesting the morphology upon mitotic entry had reached a steady state within 3.5 h of the addition of either drug. These data delineate similarities between the two drugs in their ability to form aberrant asters through a common set of phenotypically similar intermediate spindle morphologies. However, it also highlights their differences; including the fact that paclitaxel inhibits cell division at earlier timepoints while the multiple aster phenotype is observed to a greater extent after taccalonolide A treatment.

### NuMA is required for aster maturation

During evaluation of the time course images of mitotic aster formation, it was evident that several of the cells which contained multiple asters upon mitotic entry passed through an intermediate stage where the microtubules localized to cell periphery as well as internal areas that may overlap with nuclear material (Figure 3A, bottom row; Additional file 1: Figure S1). To gain higher temporal resolution of this process, images of taccalonolide A-treated cells were acquired every minute for 1 h starting 3 h after drug addition. Multiple microtubule asters were indeed found to be first nucleated at the cortical membrane in every cell that entered mitosis with multiple asters (data not shown), indicating that this is a common intermediate stage in the formation of these aberrant mitotic asters. Representative images depicting the localization of microtubules to the cell periphery and around DAPI stained nuclear material 4 h after taccalonolide A-treatment are shown in Additional file 1: Figure S1. It has been previously shown that high concentrations of paclitaxel also cause a redistribution of microtubules to the cell cortex where they co-localize with the microtubule crosslinking protein NuMA [15].

The role and requirement for NuMA in the formation of taccalonolide-induced aberrant mitotic asters was investigated. An 80% depletion of NuMA protein levels by siRNA did not affect mitotic entry in the presence or absence of taccalonolide A as determined by chromosome condensation and the RhoA-mediated rounding up of cells that occurs prior to mitotic entry (Figure 3D). Evaluation of mitotic entry as defined by these criteria indicated mitotic indices of 39% and 32% in GADPH-depleted control cells and NuMA-depleted cells, respectively. The morphology of mitotic asters in taccalonolide A-treated mitotic cells depleted of NuMA was notably different from that observed in cells expressing normal levels of NuMA. While the early taccalonolide A-induced mitotic phenotype with microtubules congregated around the cellular cortex was readily observed in NuMA-depleted cells, there was a noticeable decrease in mitotic cells with mature, fully formed asters (Figure 3D). Quantification of spindle phenotypes indicated that NuMA depletion reduced the percentage of mitotic cells containing mature, punctate asters from 25% to 12%, with a concomitant increase in mitotic cells with cortically localized microtubules. These data are consistent with previous reports describing the localization of microtubules to the cell cortex upon mitotic entry in the presence of microtubule targeting agents as a passive process [15] and suggest a role for NuMA in taccalonolide A-induced aster formation from these cortically organized microtubules.

### Differential aster resolution identified by live cell microscopy

**Figure 4:**
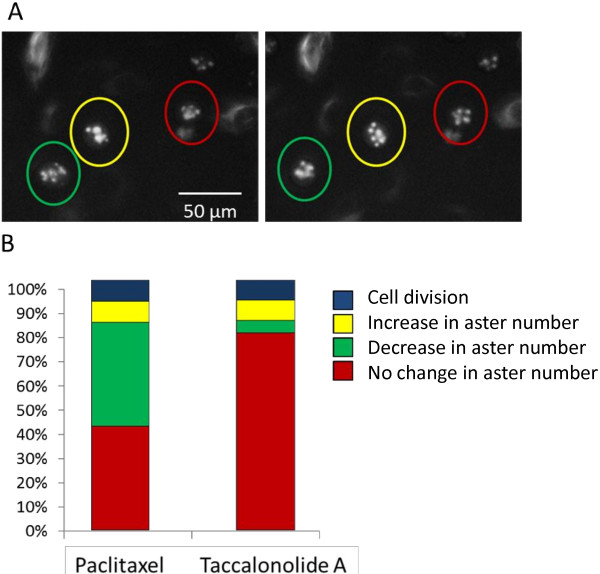
**Resolution of drug induced asters.** The resolution of the multiple asters formed in GFP-β-tubulin expressing HeLa cells after the addition of 12 nM paclitaxel or 5 μM taccalonolide A were evaluated 5–8 h after drug addition. **(A)** A representative microscope field showing mitotic cells that undergo: 1) a decrease in aster number (green circle), 2) increase in aster number (yellow circle) or 3) no change in number of asters (red circle) are indicated. **(B)** Percentage of mitotic cells that undergo each phenotype or successfully divide during the time period. 100 cells were followed over time for each condition.

The overriding similarities in aster formation for cells entering mitosis in the presence of paclitaxel or taccalonolide A were somewhat surprising given the very distinct aster morphologies observed when cells had been treated for 18 h with either drug (Figure 1). To further understand these steady-state phenotypic differences, changes in the aster morphologies of cells containing multiple asters were monitored after their initial formation. Three distinct phenotypes were observed over time as shown in Figure 4A: 1) an increase in the total number of asters (yellow), 2) a decrease in the total number of asters (green) or 3) no significant change in the number of asters (red). When these phenotypes were quantified, approximately 80% of taccalonolide A-treated cells that contained multiple asters 5 h after drug addition showed no gross changes in morphology 3 h later (Figure 4B). In contrast, almost half of paclitaxel-treated cells consolidated their asters over the same time period, resulting in fewer, larger asters. The consolidation of asters was occasionally able to resolve to the extent that cell division occurred within the observed time frame. This consolidation of asters was not uncommon in paclitaxel-treated cells, but rarely occurred with taccalonolide A treatment. These results are consistent with the differences observed in the steady-state aster morphologies after treatment with each microtubule stabilizer (Figure 1) and with the fact that fewer cells are observed in mitosis after paclitaxel treatment (Figure 2). These experiments were reproduced with the more potent taccalonolides AF and AJ and the results were consistent to those obtained with taccalonolide A, particularly with regard to the inability of asters to coalesce (data not shown), suggesting that this is a common mechanism of the taccalonolides.

## Discussion

Paclitaxel and the taccalonolides, drugs representing two classes of microtubule stabilizers, were compared for their effects on microtubule dynamic instability and mitotic aster formation. Although the steady state aster morphologies of cells treated with the taccalonolides or paclitaxel are quite distinct (Figure 1), time lapse microscopy indicated that the microtubule structures formed at the onset of mitosis are essentially identical (Figure 3). These two microtubule stabilizers did not affect the rate of mitotic entry and cells treated with either drug showed a similar evolution of three distinct mitotic fates. At 0–2 h after drug addition, cells entering mitosis continued to form a seemingly normal bipolar spindle and to complete cellular division. Once cells had been exposed to either drug for 2–3 h, the majority of cells formed bipolar spindles upon mitotic entry that either persisted in mitosis or transitioned into multiple asters. Finally, the majority of cells that entered mitosis more than 3 h after addition of either drug formed multiple asters after initially nucleating microtubules near the cell periphery. Interestingly, although NuMA depletion did not appear to affect the ability of cells to enter mitosis with nucleation of microtubules at the cell periphery (which appears to be an early step in multiple aster formation), it inhibited the ability of these cortically localized microtubules to form mature asters (Figure 3D). This finding is consistent with the ability of NuMA to nucleate microtubule asters even in cell free systems [16].

Although the initial process of aster formation in response to treatment with the two drugs is similar, the effects on the fate of these mitotic asters are distinct. The multiple asters formed in the presence of paclitaxel tend to merge together over time to form larger, more diffuse asters. This tendency for paclitaxel-treated cells to consolidate multiple asters is similar to previous study results showing that cells containing supernumerary centrosomes also consolidate them to correct the defect and form a bipolar spindle [17]. Although the multiple asters formed during treatment with microtubule stabilizers are not a result of centrosomal over duplication [14], a similar mechanism might be responsible for consolidating these non-centrosomal asters into structures that more closely resemble a bipolar spindle. The fact that paclitaxel-treated cells are able to consolidate multiple small asters into fewer, larger structures suggests that aster coalescence does not require the presence of centrosomal proteins.

In contrast to the aster consolidation that occurs with paclitaxel, asters formed in the presence of the taccalonolides are more static. In the vast majority of taccalonolide-treated mitotic cells, no changes in aster number and/or morphology were observed after their initial formation (Figure 4). This finding is intriguing in light of the observation that the taccalonolides and paclitaxel have subtle differences in their ability to inhibit parameters of microtubule dynamic instability in both purified tubulin preparations or in live cells (Tables 1, 2). While taccalonolide AJ suppressed catastrophe frequency in both assays, paclitaxel only did so in live cells where there are numerous other microtubule associated proteins that also affect microtubule dynamics (Table 2). We hypothesize that the intrinsic ability of the taccalonolides to profoundly suppress microtubule catastrophe in purified tubulin preparations may play a role in the finding that they prevent microtubule asters from coalescing into fewer, larger structures in mitotic cells. Additionally, it has recently been reported that the taccalonolides impart a dramatic inter-protofilament stability to microtubules by covalently binding to the 212–230 peptide of β-tubulin [13]. This binding may also indirectly affect microtubule dynamics in cells by altering their association with microtubule associated proteins, including molecular motors, or their ability to receive post-translational modifications.

The finding that taccalonolide AJ is less potent than paclitaxel in its ability to impact the dynamics of purified tubulin but more potent in its ability to do so in live cells is also consistent with our previous observations that the cellular effects of the taccalonolides are more persistent than paclitaxel, which may relate to their ability to be concentrated and/or retained in cells. This has been observed for other microtubule targeted agents, including paclitaxel, which are concentrated across the cellular membrane and to generate intracellular drug concentrations that are much higher than the surrounding medium [18-22]. We hypothesize that the covalent binding of the taccalonolides to tubulin/microtubules [13] also allows them to be retained and concentrated in the cell, which may allow for a higher local concentration that is sufficient to stabilize microtubules. It will be interesting to test this hypothesis with the generation of radiolabeled material. Together, these observations suggest that the taccalonolides impart increased stability to intact microtubules, as compared to paclitaxel, which may make them less prone to reorganization.

In our analysis of aster formation in the presence of microtubule stabilizers, the minimum concentrations of drugs that caused maximal mitotic arrest were used to directly compare their effects on mitotic entry (Figure 1). Another study analyzed aster formation at concentrations of paclitaxel up to three orders of magnitude higher than those that cause mitotic arrest [15]. In this earlier study, the formation of asters followed an identical mechanism; microtubules were initially localized to the cell cortex upon entry into mitosis after which they formed multiple asters. In contrast to our study using 12 nM paclitaxel, the asters that formed in the presence of 10 μM paclitaxel did not coalesce over time, which was similar to the effects observed with antiproliferative concentrations of the taccalonolides. This finding demonstrates that high concentrations of paclitaxel, several times higher than those which cause mitotic arrest, can lead to the formation of asters similar to those formed by the taccalonolides at low, antiproliferative concentrations. Indeed, we also observed that at concentrations of paclitaxel 10 – 50 times higher than those that cause mitotic arrest, asters began to have a lower propensity to coalesce. Therefore, it appears that microtubule stabilization is correlated with the concentration of drug, which is consistent with the high level of stabilization observed with a covalently bound drug such as taccalonolide AJ. However, it is also possible there is some mechanistic difference between drugs as even extremely high concentrations of paclitaxel do not cause the dramatic inhibition of aster consolidation observed with antiproliferative concentrations of the taccalonolides (unpublished observation).

## Conclusions

Together, these data strongly suggest that the taccalonolides have a distinct ability to impart microtubule stability by directly inhibiting microtubule catastrophe, which may explain some of the observed effects of this class of microtubule stabilizers, including their ability to inhibit the consolidation of microtubule asters in mitotic cells at anti-proliferative concentrations.

## Methods

### Materials

Taccalonolide A was isolated from plants of the genus *Tacca* as previously described [10]. The taccalonolide A used in this study is identical to the material used in several previous cellular based studies [4,10,23]. Taccalonolide AJ was generated by semi-synthesis as described previously [12]. Paclitaxel was obtained from Sigma (St. Louis, MO). Ethanol was used as a vehicle for all drugs.

### Cell culture

HeLa cells were purchased from American Type Tissue Culture Collection (Manassas, VA). GFP-β-tubulin expressing HeLa cells were kindly provided by Dr. Paul Chang of MIT. Cells were grown in Basal Media Eagle (Invitrogen; Carlsbad, CA) supplemented with 10% fetal bovine serum (Hyclone; Logan, UT) and 50 μg/ml gentamicin sulfate (Invitrogen). MCF7 breast carcinoma cells, stably expressing enhanced green fluorescence protein conjugated to α-tubulin (MCF7-EGFP-α-tubulin) [24] were cultured in Dulbecco’s Modified Eagle’s Medium (DMEM) (Sigma-Aldrich, St. Louis Missouri) containing 3.7 g/L sodium bicarbonate, 1% non-essential amino acids, and 1% penicillin-streptomycin, pH 7.2. Cells expressing EGFP-tubulin were selected by culturing cells in DMEM supplemented with G418 (0.5 mg/ml) for 2 weeks. Cells were used within six months of resurrection from liquid nitrogen.

### Microtubule dynamic instability with phosphocellulose purified tubulin

Effects of taccalonolide AJ or paclitaxel on the dynamic instability of phosphocellulose purified, MAP-free bovine brain microtubules were determined using differential interference contrast microscopy as previously described [25]. Briefly, tubulin (17 μM) was assembled onto the ends of sea urchin (*Strongylocentrotus purpuratus*) axoneme seeds with or without drug in PMEM buffer (87 mM Pipes, 36 mM MES, 1 mM EGTA, 2 mM MgCl_2_, pH 6.8) in the presence of 2 mM GTP. Samples were incubated for 30 min at 35ºC for the microtubules to reach steady state. Real-time, 10 min-duration videos of the microtubules were collected using an Olympus IX71 inverted microscope with a 100× oil immersion objective (NA = 1.4) at 35ºC . Microtubules were tracked using the software RTMII, and analyzed using IgorPro (Media Cybernetics, Bethesda, MD) [26]. Dynamic instability parameters were determined as described [27]. At least 25 microtubules were analyzed per condition and statistical analysis was performed using a Student’s *t*-test.

### Microtubule dynamics in cells

Drugs were added to MCF7-EGFP-α-tubulin cells in 1% FBS for 4 h after which coverslips were placed in recording media (10% FBS-DMEM, lacking phenol red and sodium bicarbonate, but supplemented with 15 mM HEPES, 3.5 g/L glucose, Oxyrase (1:50 dilution), and DL-lactate (10 mmol/L)). Cells were visualized using a 100× Nikon Plan Apo objective (N.A. 1.4, oil immersion) at 37°C on a Nikon Eclipse E800 microscope (Nikon; Tokyo, Japan) equipped with a CoolSNAP HQ2 camera (Roper Scientific GmbH, Ottobrunn, Germany). Images were taken at 4-s intervals for 2.5 min using an exposure time of 600 ms, no binning, and an 8-bit image auto scale using Metamorph software (Molecular Devices, Sunnyvale, CA) [28]. Plus ends of microtubules were tracked using Igor Pro 6.22A: Microtubule Life History Analysis Package designed by Dr. Emin Oroudjev (University of California Santa Barbara, 2010). Dynamic instability parameters were determined as described [28]. A minimum of 30 microtubules were measured from three independent experiments per condition, and reported as mean ± SEM.

### High content live cell microscopy

HeLa cells expressing GFP-β-tubulin were grown in 384-well bottom view plates to 80% confluence. Taccalonolides A, AJ or paclitaxel were added to 12 wells at the minimum concentration that caused maximal G_2_/M arrest as determined by flow cytometry. Ethanol was added to an additional 12 wells as a vehicle control. Images from 5 frames in each of the 12 wells for each condition were acquired every 30 min for 8 h using the 20× long working distance objective of the Operetta high content imaging system (PerkinElmer, Waltham, MA). Mitotic cells in each frame were identified and the number of mitotic asters in each cell was counted at each time point. A total of 60 images were evaluated at each time point for each treatment condition. The time when individual cells entered mitosis was identified using these images. Entry into mitosis was defined by the clear transition from an interphase microtubule cytoskeleton to one containing mitotic spindles or asters between two consecutively acquired images. Time-lapse movies for each field were then made from the images to determine the changes that occurred in the spindle/aster morphology of individual cells over time.

### Immunofluorescence microscopy and NuMA siRNA

HeLa cells were transfected with NuMA or GADPH siRNA (Life Technologies, Grand Island, NY) in oligofectamine for 72 h. Taccalonolide A was added to for 4 h after which cells were immediately fixed and microtubules visualized by indirect immunofluorescence for β-tubulin with a Nikon Eclipse 80i fluorescence microscope and NIS Elements software as previously described [29]. The distribution of cells in interphase, in mitosis with cortically localized tubulin, and in mitosis with asters were determined. A minimum of 800 cells were evaluated for each treatment condition.

## Competing interests

Authors Risinger and Mooberry are inventors on a pending patent application on new taccalonolides that is assigned to the University of Texas system.

## Authors’ contributions

ALR conceived of the study and design, carried out experiments pertaining to aster formation and resolution, prepared figures and drafted the manuscript. SMR and ML performed microtubule dynamics experiments, analyzed the data and helped to draft the manuscript. LW, MAJ and SLM participated in study design, data analysis and drafting of the manuscript. All authors read and approved the final manuscript.

## Supplementary Material

Additional file 1: Figure S1 Microtubules localized to the cell periphery and nuclear region during early aster formation. Microtubules in HeLa cells treated for 4 h with 5 μM taccalonolide A were visualized by indirect immunofluorescence for β-tubulin (green). Cells undergoing early stages of aster formation with microtubules localized to the cell periphery and near DAPI stained nuclear material (blue) are indicated with arrows. Click here for file
