# Supplementary figures and images for: The taccalonolides and paclitaxel cause distinct effects on microtubule dynamics and aster formation
Source: Mol Cancer. 2014 Feb 28;13:41. doi: 10.1186/1476-4598-13-41 (PMC4015978; doi:10.1186/1476-4598-13-41)

## Slide 1
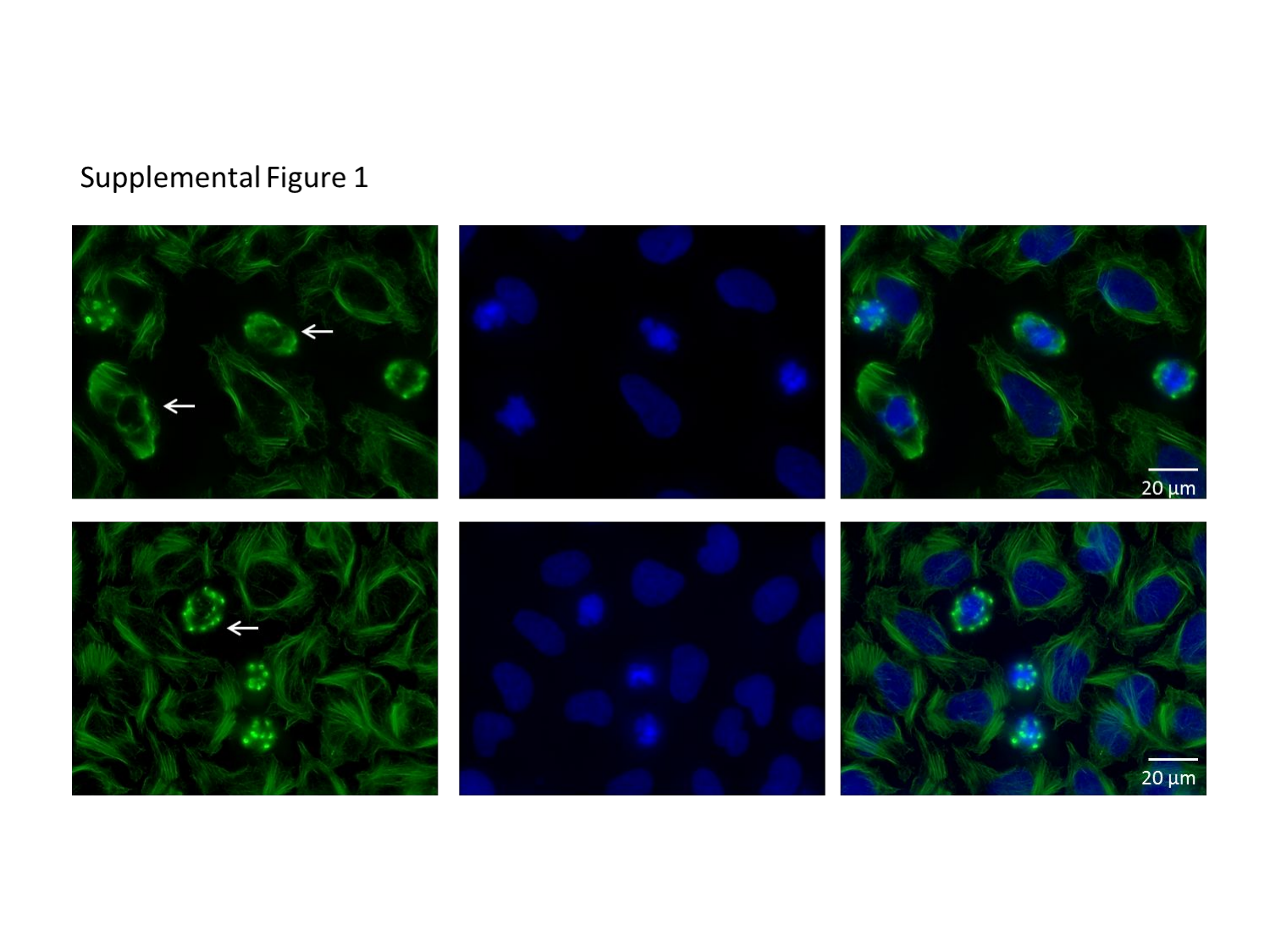

Supplement: Additional file 1: Figure S1 — Microtubules localized to the cell periphery and nuclear region during early aster formation. Microtubules in HeLa cells treated for 4 h with 5 μM taccalonolide A were visualized by indirect immunofluorescence for β-tubulin (green). Cells undergoing early stages of aster formation with microtubules localized to the cell periphery and near DAPI stained nuclear material (blue) are indicated with arrows. [file 1476-4598-13-41-S1.pptx]
